# Immobilized Enzymes for ABO‐Independent Blood Cell Transfusions

**DOI:** 10.1002/cbic.202500542

**Published:** 2025-11-23

**Authors:** Christina Möller, Fabian Pohlschröder, Jan Wesche, Paula Schmiade, Merle Baselau, Kristian Wende, Berenika Stloukalová, Dominique Böttcher, Andreas Greinacher, Uwe T. Bornscheuer, Henrik Terholsen, Konstanze Aurich

**Affiliations:** ^1^ Department of Biotechnology and Enzyme Catalysis Institute of Biochemistry University of Greifswald Felix‐Hausdorff‐Straße 4 17487 Greifswald Germany; ^2^ Institute of Transfusion Medicine University Medicine Greifswald Fleischmannstraße 8 17475 Greifswald Germany; ^3^ Leibniz Institute for Plasma Science and Technology Felix‐Hausdorff‐Straße 2 17489 Greifswald Germany; ^4^ Present address: Department of Chemical and Pharmaceutical Biology Faculty of Science and Engineering University of Groningen Antonius Deusinglaan 1 Groningen 9713 AV The Netherlands

**Keywords:** biocatalysis, blood type antigens, enzyme immobilization, glycoside hydrolases, universal blood

## Abstract

The transfusion of blood products requires ABO compatibility due to A and B antigens on blood cells and their corresponding antibodies in plasma. The supply of red cell concentrates (RCCs) and platelet concentrates (PCs) is challenged by rising demand and declining numbers of donors. To counteract the supply limitations, a method is developed to generate universal RCCs and PCs. For this, the A antigen is enzymatically removed by two enzymes from *Flavonifractor plautii* working in concert and the B antigen is removed by galactosidases from *Pedobacter panaciterrae* (PpaGal_WT or the variant PpaGal_W260Y) and *Akkermansia muciniphila* (AmGH110A or AmGH110B). The glycosidases are immobilized on polymethacrylate microparticles and are removed by the 200 µm filter of the mandatory transfusion set before the transfusion to avoid a possible immune reaction by the enzymes. B‐positive red cells in RCCs are reduced below a 4% background signal within 48 h at 2–6 °C. Pooled PCs showed a 34 ± 14% reduction in B‐positive cells and a residual A‐positive platelet population of 4 ± 1% after 4 h of treatment at 20–24 °C. This approach efficiently generates ABO‐universal RCCs and PCs while preserving blood quality and reducing incompatibility risks.

## Graphical abstract

A method is developed to generate universal red blood cells and platelets using suitable glycosidases immobilized to polymethacrylate microparticles. This approach allows the efficient removal of the enzymes converting the A and B antigens before transfusion to avoid immune reactions and illustrates a promising method to counteract the demand for blood products.

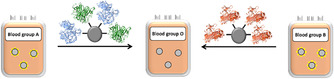

## 1 Introduction

**Figure 1:**
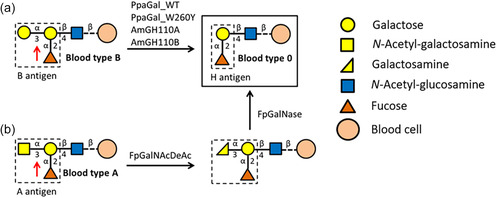
Schematic overview of enzymatic antigen removal a) of the B antigen by *α*−1,3‐galactosidases from *Pedobacter panaciterrae* (PpaGal_WT or PpaGal_W260Y) and *Akkermansia municiphila* (AmGH110A or AmGH110B) and b) of the A antigen by *N*‐acetyl‐*α*‐D‐galactosamine deacetylase (FpGalNAcDeAc) and *α*‐d‐galactosaminidase (FpGalNase) from *Flavonifractor plautii*. Sugars are shown using the Consortium for Functional Galycomics notation.^[^ ^7^ ^]^

Transfusion requires compatibility within the ABO blood group system. This system is based on the presence of the two antigens A and B on blood cell surfaces. All blood cells contain the fucosyl galactose H antigen on their surface, determining blood type O. In blood types A and B, an additional *N*‐acetylgalactosamine (GalNAc) or galactose (Gal) is attached to the fucosyl Gal H antigen, respectively (**Figure** **1**a,b).^[^ ^1^ ^]^

The concept of enzymatic removal of ABO antigens was first demonstrated for red blood cells (RBCs)^[^ ^2^ ^]^ and the feasibility of transfusing these enzymatically generated “universal red cells” has been shown in phase 1 and 2 clinical trials.^[^ ^3^, ^4^, ^5^ ^]^ Enzyme specificity and efficacy for the removal of the A and B blood groups have been improved in follow up studies.^[^ ^6^, ^7^, ^8^ ^]^ B antigen removal was shown to be enhanced using *α*−1,3‐galactosidases from *Akkermansia muciniphila*, a mucin‐degrading gut bacterium.^[^ ^9^ ^]^ We also recently identified a more efficient GH110 galactosidase from *Pedobacter panaciterrae* (PpaGal_WT), and further improved its activity through protein engineering (PpaGal_W260Y, Figure 1a).^[^ ^10^ ^]^ For the conversion of the A antigen a *N*‐acetyl‐*α*‐D‐galactosamine deacetylase (FpGalNacDeAc) and *α*‐D‐galactosaminidase (FpGalNase) from *Flavonifractor plautii* efficiently work in concert.^[^ ^7^ ^]^ Besides enzyme efficacy, the potential immunogenicity and adverse immune reactions to these enzymes in the transfusion recipient are a major concern. Removal of the glycosidases is currently achieved by intensively washing the blood product.^[^ ^7^ ^]^ However, this process is time‐consuming and may compromise blood cell integrity.

The transfusion of blood has evolved from the transfusion of whole blood to the transfusion of blood products, including red cell concentrates (RCCs), platelet concentrates (PCs), and plasma, allowing the treatment of several patients from one whole blood donation. However, there is a growing demand of blood products due to the demographically aging population, a decrease in donor numbers and an increase in blood‐intensive procedures.^[^ ^1^ ^,^ ^11^ ^,^ ^12^ ^]^ While RCCs are obtained from a single donor, PCs are obtained by pooling so‐called buffy coats that contain platelets, red cells, and leukocytes after centrifugation from 4 to 5 whole blood donations. Platelets are then separated by centrifugation. These pooled PCs contain 30–100% plasma, depending on the manufacturing method. The remaining anti‐A and anti‐B antibodies in the plasma still require an ABO identical PC transfusion. We have recently developed a method to remove anti‐A and anti‐B from therapeutic plasma.^[^ ^13^ ^]^

Here, we describe an approach for producing ABO‐independent universal RCCs and PCs through the removal of A and B antigens using A‐ and B‐specific glycosidases immobilized on polymethacrylate microparticles, along with the elimination of residual plasma anti‐A and anti‐B antibodies. In addition, we provide a new, easy, and efficient approach to overcome the problem of potential immunogenicity of residual glycosidases in the blood product.

## 2 Results and Discussion

Universal blood products eliminate the need for ABO blood type matching. This helps to reduce transfusion delays, improve emergency preparedness, and ensure broader availability for patients with rare or unknown blood types. Although pathways for universal human plasma using isoagglutinin binding and removal are established,^[^ ^13^ ^,^ ^14^ ^]^ a suitable approach for cellular blood products that results in transfusable universal blood products is missing.

### 2.1 Enzyme Immobilization on Microparticles

**Figure 2:**
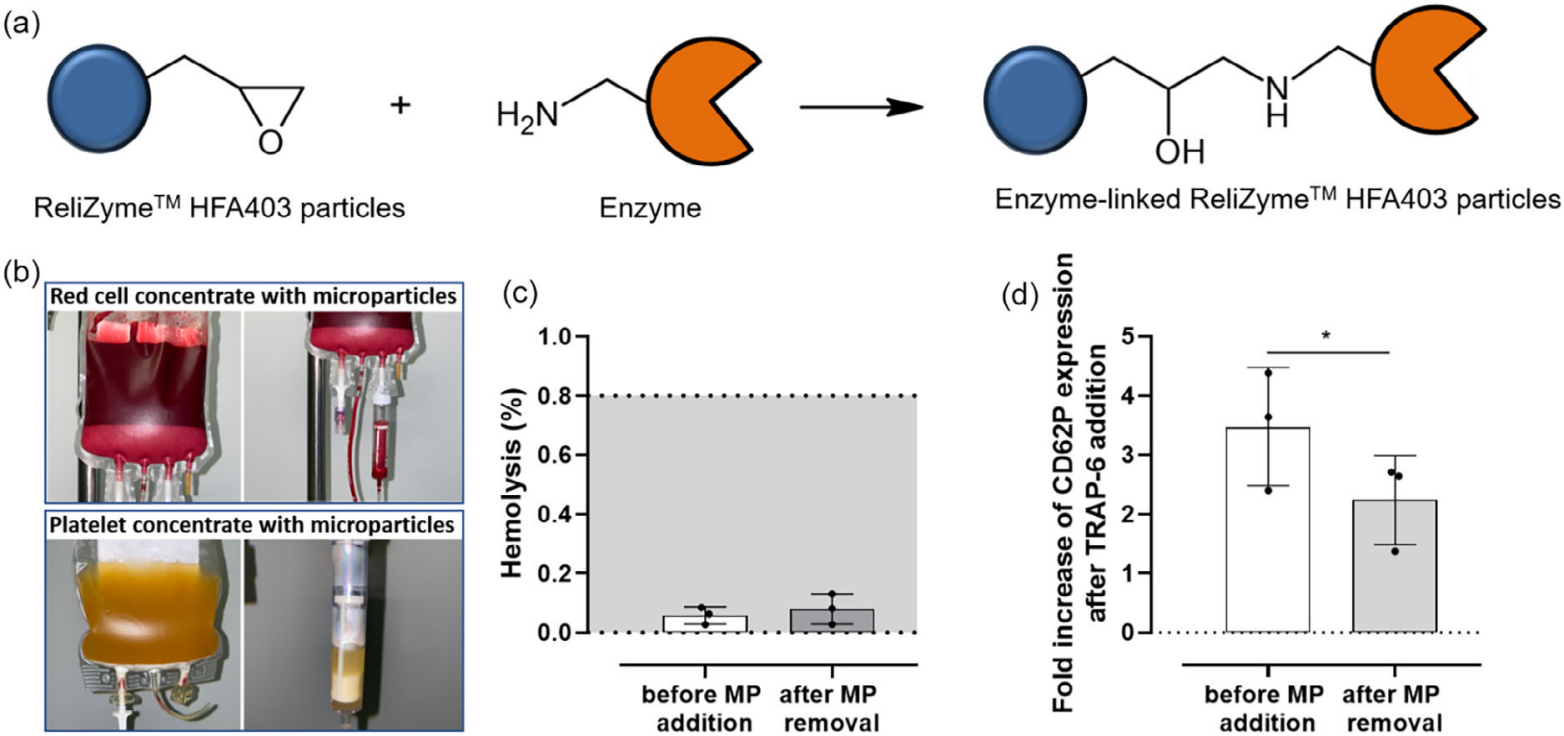
ReliZyme HFA403 microparticles as solid phase for enzyme binding. a) Scheme of covalent enzyme binding on epoxy functionalized ReliZyme HFA403 microparticles via primary amino groups and b) enzyme‐coupled ReliZyme HFA403 microparticles are removed by the transfusion set filter in RCCs and PCs. c) No hemolysis is induced by ReliZyme HFA403 microparticles (10 mg . 2 mL^−1^) on red cells (dotted line =  hemolysis rate limit of red cell concentrates according to European guidelines for blood =  0.8%^[^ ^32^ ^]^) and d) platelet functional capacity remains preserved in the presence of ReliZyme HFA403 MP in platelet concentrates and is slightly reduced after the removal procedure; determined as fold increase of CD62P expression after TRAP‐6 addition (40 µM). *n* =  3, mean ± standard deviation. Normality was assessed with the Shapiro–Wilk test. Group differences were analyzed using one‐way repeated‐measures ANOVA (Geisser33–Greenhouse correction) with Fisher's LSD for pairwise comparisons, or the Friedman test for non‐normal data. *p < 0.05.

We developed a method to produce universal RCCs and PCs by removing blood group antigens with solid‐phase bound enzymes, followed by their complete removal in a closed bag system. Covalent attachment of the enzymes to epoxy‐functionalized ReliZyme HFA403 microparticles (MP) occurred via their primary amino groups (**Figure** **2**a). We bound the enzymes on MP at 20–24 °C with an overnight incubation at the physiological blood pH of 7.4. Maintaining a moderate temperature upon covalent immobilization on beads might enhance enzyme flexibility and nucleophilic reactivity, improving the binding efficiency.^[^ ^15^ ^]^ The addition of 3 M ammonium sulfate increases ionic strength, neutralizes enzyme surface charge, and promotes stronger hydrophobic interactions with the used MPs. This led to fast and efficient binding, enhancing enzyme concentration on the surfaces of the MPs.^[^ ^16^ ^]^ We achieved a binding of 78% of the A antigen removing enzymes FpGalNAcDeAc and FpGalNase (6 µM each) and of 90% of the representative B antigen removing galactosidase PpaGal_WT (11.8 µM) after an overnight incubation (Figure S1, Supporting Information). For A antigen removing enzymes, we coimmobilized the *N*‐acetyl‐*α*‐D‐galactosamine deacetylase (FpGalNAcDeAc) and the *α*‐D‐galactosaminidase (FpGalNase) from *Flavonifractor plautii* in a one‐step procedure. Coimmobilization can increase the catalytic efficiency of enzymes acting in sequence by drastically reducing the diffusion pathways and diffusion times to the substrate.^[^ ^17^ ^,^ ^18^ ^]^

To avoid possible immune reactions by the enzymes, the glycosidases have to be efficiently removed. MPs with the immobilized enzymes were completely removed using 200 µm filters of standard transfusion sets (Figure 2b). Their diameter (200–500 µM) safely exceeds the pore size of standard transfusion filters, allowing for efficient removal during transfusion, thereby streamlining the process while maintaining product safety and functionality. To evaluate the influence of potential shear forces generated by the particles, we analyzed RBC hemolysis—a key quality parameter of RCC—both before MP addition and after their removal via the transfusion set (Figure 2c). Platelet functional capacity, assessed by CD62P expression following thrombin receptor activating peptide (TRAP)−6 stimulation, showed only slight impairment after MP removal (Figure 2d).

### 2.2 Producing Universal RBCs by Enzymatic Removal of B Antigens

**Figure 3:**
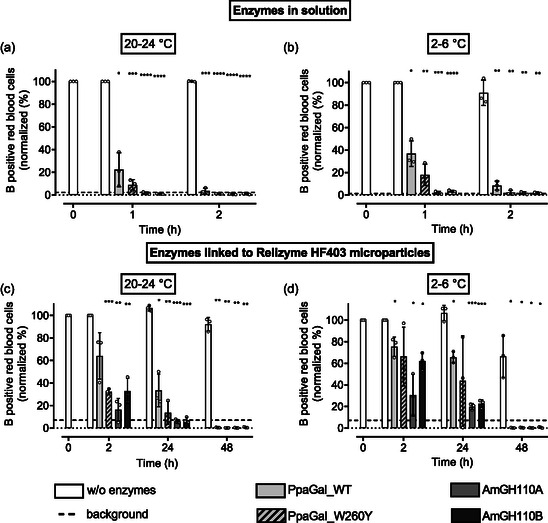
Enzymatic removal of B antigen from RBCs. B antigen removal from RBCs by galactosidases from *Pedobacter panaciterrae* wild type (PpaGal_WT) and engineered (PpaGal_W260Y) and galactosidases from *Akkermansia muciniphila* A (AmGH110A) and B (AmGH110B) in solution (each 1.18 µM) a) at 20–24 °C, b) at 2–6 °C; and linked to ReliZyme HFA403 microparticles c) at 20–24 °C and d) at 2–6 °C. *n* =  3, mean ± standard deviation. Normality was assessed with the Shapiro–Wilk test. Group differences were analyzed using one‐way repeated‐measures ANOVA (Geisser33–Greenhouse correction) with Fisher's LSD for pairwise comparisons, or the Friedman test for non‐normal data. *p < 0.05, **p < 0.01, ***p < 0.001, and ****p < 0.0001. Differences are indicated compared to incubation without enzymes.

Since the enzymatic removal of the A antigen by FpGalNAcDeAc and FpGalNase from RBCs is well‐established,^[^ ^7^ ^,^ ^18^ ^]^ we focused on enzymatic B antigen removal. Here, we used our newly engineered galactosidase variant, PpaGal_W260Y,^[^ ^10^ ^]^ alongside wild‐type PpaGal (PpaGal_WT) and the recently described galactosidases from *Akkermansia muciniphila* AmGH110A and AmGH110B.^[^ ^9^ ^]^ A 1 h incubation at 20–24 °C effectively reduced B‐positive RBCs below background levels using AmGH110A and AmGH110B (2 ± 1% and 1 ± 0%, respectively **Figure** **3**a). PpaGal_W260Y and PpaGal_WT were less efficient, yielding 9 ± 5% and 22 ± 15% of B‐positive RBCs, respectively. Lowering the incubation temperature to the storage temperature of RCCs 2–6 °C produced similar results across all four galactosidases (Figure 3b). The higher efficiency of AmGH110A and AmGH110B might be explained by the fact that the amino acids of the surface close to the active site of these enzymes are rather polar, which might enhance the interaction with the surface of RBCs and platelets that is negatively charged due to the presence of the carboxyl group of sialic acids in the cell membrane.^[^ ^19^ ^]^ Moreover, AmGH110A contains an additional N‐terminal carbohydrate‐binding module CBM51, which specifically acts on B antigens and therefore might further enhance the enzymes´ activity.^[^ ^9^ ^,^ ^20^ ^]^ Finally, the amino acid corresponding to position 260 in the galactosidase of PpaGal is naturally occupied by a tyrosine in AmGH110A and AmGH110B and may contribute to improved activity. We recently demonstrated that substituting this residue, as in PpaGal_W260Y enhances B antigen processing activity by 2.5‐fold compared to the wild type, suggesting a functional advantage conferred by tyrosine at this position.

When immobilized on ReliZyme HFA403 MPs, the efficacy of the enzymes was lower; however, after 48 h incubation of RBC with the different galactosidases, B‐positive RBCs were decreased below the background signal under all given conditions. After 24 h at 20–24 °C, microparticle‐bound AmGH110A and AmGH110B reduced B‐positive RBCs below background levels. The addition of microparticle‐bound PpaGal_W260Y resulted in a B‐positive cell percentage of 26 ± 20%, and PpaGal_WT of 30 ± 18% (Figure 3c). Despite the optimization of the immobilization conditions, antigen removal by immobilized enzymes is slower than by the free enzymes in solution. A possible reason for this could be the inaccessibility of the enzyme´s active site after immobilization, for instance, if the active site faces the particle surface. This is plausible since the immobilization technique used is not site‐directed. Another explanation could be multipoint immobilization, which might restrict the enzyme´s conformational flexibility and thereby impair its function. Additionally, the attack surface for the antigen located on the blood cell surface might be too small. The short linkers on the surface of the functionalized MP and the use of porous MP might contribute to this problem. Since RCCs are stored at 2–6 °C up to 49 days, ensuring efficient antigen removal under these storage conditions would be beneficial to maintain the mandatory cold chain. Even though the enzymatic cleavage was slowed down at 2–6 °C, we successfully removed blood type B antigens within 48 h with all the used galactosidases (Figure 3d).

### 2.3 Enzymatic Removal of Blood Group Antigens from Platelets

Unlike RCCs, which only contain material from one donor, PCs are obtained by pooling platelets from buffy coats of 4–5 donors of identical blood groups (pooled PCs) and are stored up to seven days. Blood shortages often result in fewer than 4–5 buffy coats of the same blood group being available to produce a PC. To overcome this issue, we aimed to produce a pooled PC from buffy coats of nonidentical ABO blood groups.

First, we removed ABO blood group antigens on the platelet surface by enzymatic deglycosylation as described above for RBCs. For the enzymatic conversion of the A antigen to the H antigen, we again selected FpGalNAcDeAc and FpGalNase (each 6 µM). For the enzymatic removal of B antigens, we used the four galactosidases AmGH110A, AmGH110B, PpaGal_WT, and PpaGal_W260Y (1.18 µM). The enzymes were incubated with platelets from PCs of the corresponding blood type.

**Figure 4:**
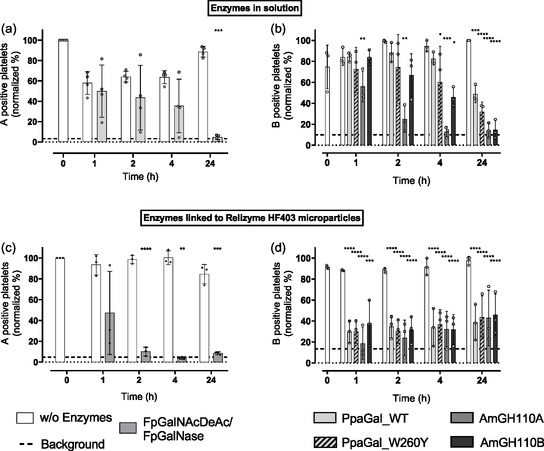
Enzymatic removal of A and B blood group antigens from platelets. Blood group antigens are removed from platelets after the addition of a) FpGalNAcDeAc (56 nM) and FpGalNase (80 nM) to blood group A platelet concentrates (PCs) and b) *α*−1,3‐galactosidase from *Pedobacter panaciterrae* (PpaGal_WT and PpaGal_W260Y, 1.18 µM) and from *Akkermansia muciniphila* (AmGH110A and AmGH110B, 1.18 µM) to blood group B PCs. Enzymes linked to ReliZyme HFA403 microparticles are able to remove blood group antigens on platelets of c) blood group A PCs (with 6 µM FpGalNAcDeAc and FpGalNase used for immobilization) and d) blood group B PCs (with 11.8 µM of each galactosidase used for immobilization). *n* =  3, mean ± standard deviation. Normality was assessed with the Shapiro–Wilk test. Group differences were analyzed using one‐way repeated‐measures ANOVA (Geisser33–Greenhouse correction) with Fisher's LSD for pairwise comparisons, or the Friedman test for non‐normal data.**p < 0.01, ***p < 0.001, and ****p < 0.0001. Differences are indicated compared to incubation without enzymes.

The proportion of A antigen‐positive platelets was successfully decreased below the background signal within 24 h (**Figure** **4**a). The used concentrations are ten times lower than previously used for RBCs,^[^ ^7^ ^]^ as expected, given that platelets express fewer blood type antigens than RBCs.^[^ ^21^ ^]^ For the B antigen‐removing enzymes, all four used galactosidases reduced the B‐positive platelets over time. AmGH110A and AmGH110B were most effective and reduced B‐positive platelets to 14 ± 7% and 15 ± 9%, respectively (Figure 4b). After 24 h, PpaGal_WT reduced B‐positive platelets to 49 ± 8%, while the engineered PpaGal_W260Y achieved a reduction to 32 ± 9%.

When we used immobilized enzymes on the MPs, they also cleaved the A and B antigens from platelet surfaces (Figure 4c,d). The percentage of A positive platelets was decreased to 4 ± 1% after 4 h of incubation with A enzymes‐linked MPs, while B‐positive platelets were decreased to 39 ± 17% (PpaGal‐WT), 44 ± 20% (PpaGal_W260Y), 43 ± 27% (AmGH110A), and 46 ± 20% (AmGH110B) after 24 h by MP‐ linked galactosidase.

While microparticle‐immobilized galactosidases removed B antigens effectively from RBCs, they were only moderately effective on platelets. This difference might arise from the distinct membrane characteristics of each cell type. RBCs have a static, smooth membrane with uniformly exposed glycans, facilitating efficient enzyme access. In contrast, platelets possess a complex, dynamic membrane architecture, including ruffling, pseudopodia, and an open canalicular system, that limits enzyme contact with surface antigens. Moreover, platelet glycoproteins often carry highly branched glycans that sterically hinder enzymatic cleavage. These structural and dynamic features likely reduce the catalytic efficiency of immobilized enzymes on platelets.^[^ ^22^ ^]^

### 2.4 Production of Pooled Platelet Concentrates from Buffy Coats of Nonidentical ABO Blood Groups

**Figure 5:**
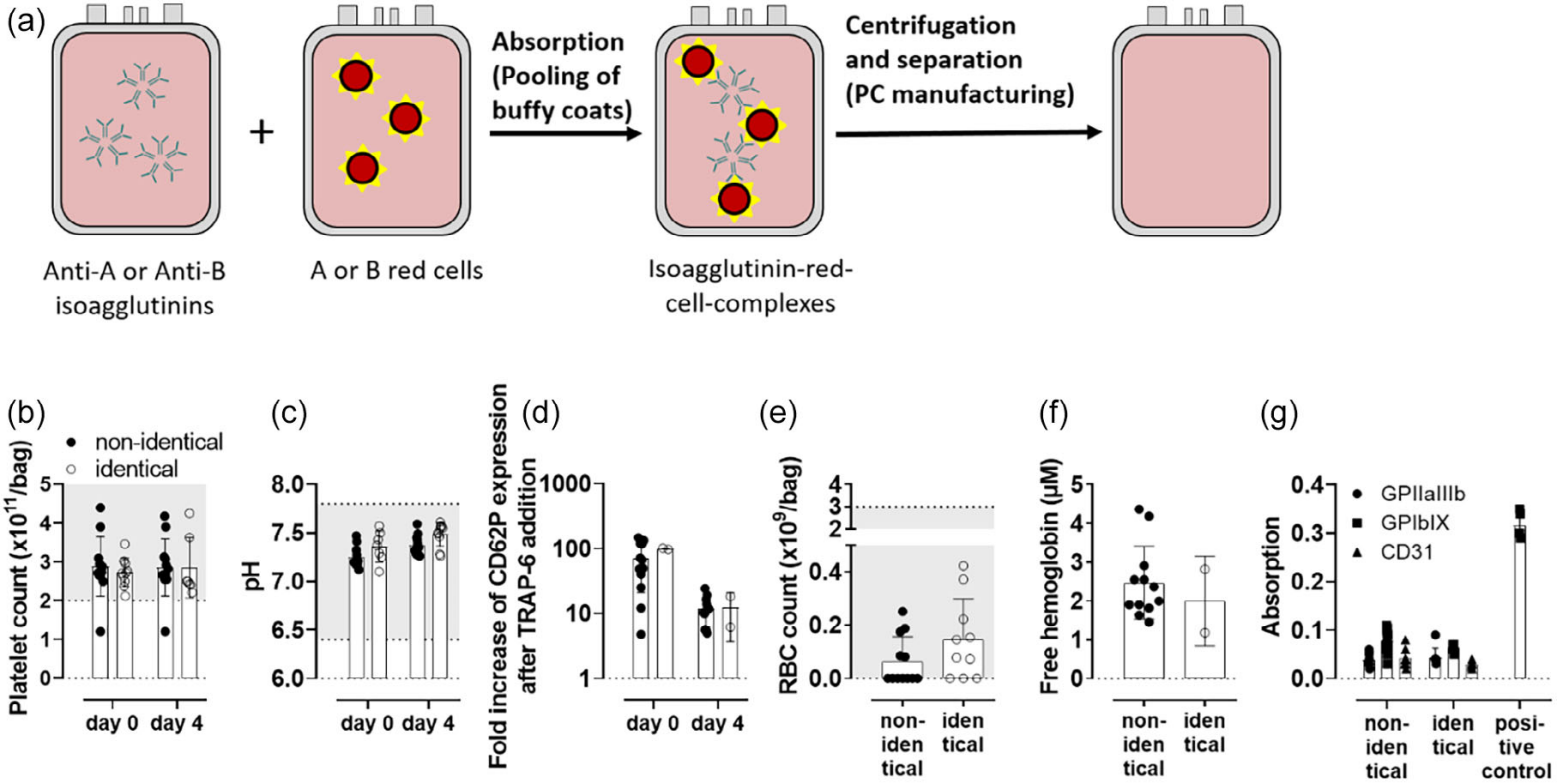
Removal of anti‐A and anti‐B antibodies in platelet concentrates (PCs) via contact with red blood cells. a) During the pooling process of buffy coats of nonidentical blood types isoagglutinins anti‐A and anti‐B are adsorbed on red cells and are removed. Comparison of pooled PCs from buffy coats of nonidentical and identical blood groups regarding b) platelet count, c) pH value, d) platelet function determined as fold increase of CD62P expression after TRAP‐6 addition (40 µM), e) red blood cell count, f) free hemoglobin, and g) antibody binding on glycoprotein IIbIIIa, glycoprotein IbIX, CD31. *n* =  12, mean ± standard deviation, all n.s. between identical and nonidentical blood groups.

We produced pooled PCs from four ABO‐nonidentical buffy coats (Table S1, Supporting Information). The A‐ and B‐RBC in the buffy coats adsorb the corresponding anti‐A and anti‐B antibodies in the remaining plasma, and the isoagglutinin‐RBC complexes are then removed by centrifugation as part of the manufacturing process,^[^ ^13^ ^]^ because RBC‐isoagglutinin complexes sediment while platelets remain in the supernatant at the centrifugation forces (**Figure** **5**a).

The regulatory quality parameters of PCs, like platelet count, pH, and preserved platelet activation after addition of thrombin receptor‐activating peptide‐6 (TRAP‐6), are comparable between PC of blood group identical and nonidentical buffy coats for up to storage day 4 (Figure 5b–d). Adsorption of anti‐A and anti‐B IgM on RBC did not cause an increase in free hemoglobin or RBC count (Figure 5e,f). We also excluded increased anti‐A and/or anti‐B IgG binding on platelet surface proteins expressing A and B blood groups, i.e., glycoprotein (GP) IIIbIIa, GP IbIX, and PECAM‐1 (CD31) by the gold standard monoclonal antibody immobilization of platelet antigens (MAIPA) assay (Figure 2g).^[^ ^23^ ^]^ Herewith, we have solved the second issue of producing universal platelets, i.e., removing the anti‐A/anti‐B antibodies from the PCs without a loss in product quality.

Due to its short shelf life, PC supply is often limited, leading to frequent ABO‐incompatible transfusions. The reduced survival of ABO‐incompatible platelets in the recipient, resulting in transfusion refractoriness, is caused by recipient isoagglutinins reacting with blood group antigens on the platelet surface.^[^ ^24^ ^]^ Repeated transfusions of nonidentical PCs can also trigger new antibody formation.^[^ ^25^ ^]^ PCs stored in 100% plasma, particularly those with elevated isoagglutinin titers, can cause hemolysis of recipient RBCs and potentially affect platelets due to minor incompatibilities.^[^ ^26^, ^27^, ^28^ ^]^ Our method of pooling buffy coats from nonidentical blood groups in platelet additive solution (PAS), followed by A/B antigen removal, addresses these challenges. Major incompatibility, which affects the platelet survival, is prevented by enzymatic antigen removal, while minor incompatibility from isoagglutinins is mitigated through adsorption onto residual buffy coat RBCs and storage in PAS. Our findings confirm that this approach preserves pooled PC quality, providing a viable solution for universal PCs.

### 2.5 Safety of Enzyme‐Coated MPs

In order to evaluate the safety profile for the enzyme‐coated ReliZyme HFA403 MP during application in a medicinal drug, we aimed to investigate possible leakage of enzymes from the MPs. The liberated enzymes could cause an immune response when given to a patient. By mass spectroscopy (Figure S2–S5, Supporting Information) only traces of FpGalNAcDeAc of 0.6 ± 0.1 µg mL^−1^ (0.2% of the bead‐bound enzyme), translating into a single digit nM concentration (Table S2, Supporting Information) and no leakage of FpGalNase and the galactosidase representative PpaGal were detected after 48 h of incubation at 20–24 °C. The number of enzyme molecules released from the microparticles into the blood bags was therefore considered negligible.

Our results indicate that incubation with ReliZyme HFA403 particles does not compromise the quality of platelet or RCCs. While direct detection of polymethacrylate leaching was not performed, no adverse effects were detected in standard quality parameters, suggesting that the risk of polymer release into the blood products under the applied conditions is minimal. Furthermore, such polymers are commonly used in tissue engineering and are known to be biocompatible, even for long‐term applications.^[^ ^29^, ^30^, ^31^ ^]^

**Figure 6:**
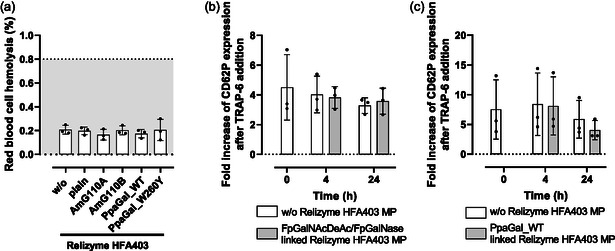
Enzyme‐linked ReliZyme HFA403 particles preserve blood cell function. a) Hemolysis of RBCs incubated with ReliZyme HFA403 particles without or bound to different galactosidases after incubation of 48 h, dotted line =  hemolysis rate limit of red cell concentrates according to European guidelines for blood =  0.8%;^[^ ^32^ ^]^ b) impact of the addition of ReliZyme HFA403 microparticles linked the A antigen‐removing enzymes from *Flavonifractor plautii* FpGalNAcDeAc and FpGalNase and c) linked to representative *α*−1,3‐galactosidase from *Pedobacter panaciterrae* (PpaGal) on platelet activation ability determined as fold increase of the CD62P expression after 40 µM thrombin receptor‐activating peptide‐6 (TRAP‐6) addition; each *n* =  3, mean ± standard deviation. All n.s.

We assessed RBC hemolysis after incubating RBCs with enzyme‐coated ReliZyme HFA403 MP at 2–6 °C for 48 h (**Figure** **6**a). Hemolysis levels were not elevated compared to RBCs without ReliZyme HFA403 MP and were well below the threshold set by the European guide to the preparation, use and quality assurance of blood components (0.8%).^[^ ^32^ ^]^

Likewise, incubation of pooled PCs with enzyme‐linked ReliZyme HFA403 MP fully preserved platelet function, determined as the fold increase of CD62P expression before and after addition of 40 µM inductor TRAP‐6 (Figure 6b,c).

## 3 Conclusion

We developed an easy and safe method to generate universal blood products. To avoid an immune response by the added enzymes, we aimed to remove them after antigen conversion by immobilization to ReliZyme HFA403 microparticles, which are then eliminated entirely by the transfusion set. Although immobilized galactosidases showed decreased activities compared to enzymes in solution, they successfully reduced B antigen‐positive RBCs and therefore can be implemented in the production process. Moreover, we could show that the concept of antigen removal can also be applied to platelets. This approach facilitates the production of universal RBC concentrates and PCs, addressing logistical challenges while maintaining quality and reducing incompatibility risks.

## 4 Experimental Section

4.1

4.1.1

##### Manufacturing of Red Cell Concentrates

Whole blood was collected from healthy donors in accordance with German hemotherapy guidelines, with written informed consent. Whole blood in citrate phosphate dextrose solution (CPD, Macopharma, Tourcouring, France) was centrifuged at 4000 × g for 10 min, and separated into RCC, plasma, and buffy coat. The RCC was leukocyte‐depleted (LCRD2‐Filter, Macopharma) and stored in phosphate‐adenine‐glucose‐guanosine‐saline‐mannitol (PAGGS‐M) solution at 2–6 °C for 49 days.

##### Manufacturing Pooled Platelet Concentrates

Buffy coats of four donations were pooled (total ≈280 mL) and 250 mL PAS E (PAS‐E, SSP+, Macopharma) was added. By centrifugation (720 × g, 15 min) platelets were separated from residual RBCs and leukocyte‐depleted (LEUCOFLEX LXT Filter, Macopharma). PC bags were stored under agitation at 20–24 °C for 4 days.

##### Quality Control of Pooled Platelet Concentrates

For PC quality control platelet count, function, residual RBCs count, free hemoglobin, antibody binding on platelets and isoagglutinin titers as quality parameters were determined (for details see the Supporting information method section).

##### Protein Production and Purification

Synthetic genes for the different galactosidases to investigate B antigen cleavage as well as two synthetic genes for the enzymatic removal of A antigens were ordered in a pET28(a) vector (BioCat GmbH, Heidelberg, Germany). All details of recombinant protein production and purification are described in the Supporting information method section.

##### Blood Group Antigen Determination on Blood Cells

The effect of enzyme addition to blood group antigens on the blood cell surface was determined by measuring the percentage of antigen A‐ and B‐positive platelets or B‐positive RBCs by flow cytometry. Please see the Supporting Information method section for details.

##### Enzyme Immobilization on Microparticles

Enzymes were immobilized covalently on polymethacrylate ReliZyme HFA 403 size M 200–500 µm microparticles (Resindion S.r.l., Italy). Please see the Supporting Information method section for details.

##### Incubation of Blood Cells with Enzymes in Solution or Linked to ReliZyme HFA403 Microparticles

*Red blood cells*. RBC from RCC were incubated with PpaGal_WT, PpaGal_W260Y, AmGH110A, and AmGH110B in solution in a final concentration of 1.18 µM each either at 20–24 °C or 2–6 °C for max. four hours. Incubation of RCC with enzyme linked ReliZyme HFA403 microparticles was performed with 20 mg enzyme‐linked MP mL^−1^ RCC for max. 48 h either at 20–24 °C or 2–6 °C. Blood bags were gently inverted twice daily to ensure distribution.

*Platelets.* Platelets from PCs were incubated with *N*‐acetyl‐*α*‐d‐galactosamine deacetylase (FpGalNAcDeAc) and *α*‐d‐galactosaminidase (FpGalNase) final concentrations of 56/80 nM or PpaGal_WT, PpaGal_W260Y, AmGH110A, or AmGH110B in solution each final concentration 1.18 µM for max 2 h at 20–24 °C. Incubation of PCs with enzyme‐linked ReliZyme HFA403 microparticles was performed with 20 mg enzyme linked MP mL^−1^ PC for max. 48 h either at 20–24 °C. During incubation, PCs were stored under agitation.

##### Impact of Enzyme‐Linked Microparticles on Blood Cells

*Red blood cells.* The impact of the microparticle addition to RCC was determined by hemolysis evaluation. Hemolysis rate of RCC was calculated from total RCC hemoglobin and free hemoglobin in the supernatant after centrifugation (4000 × g, 10 min). Total hemoglobin was determined by Sysmex XP300 analyzer (Sysmex Deutschland GmbH, Norderstedt, Germany). For free hemoglobin analysis please see the Supporting Information method section.

*Platelets.* The impact of the microparticle addition to pooled PCs was determined by the CD62P expression by flow cytometry as described in detail in the Supporting Information method section and Figure S6, Supporting Information.

##### Determination of Enzyme Residues in Platelet Concentrate Supernatant by Mass Spectrometry

Potential leakage of enzymes from beads into PC supernatant was determined using nano‐liquid chromatography/tandem mmass spectrometry (LC‐MS/MS). The method is described in detail in the Supporting Information method section.

##### Statistical Analysis

Results are presented as mean ± standard deviation (SD), unless otherwise stated. The sample size (*n*) for each analysis is indicated in the respective figure legends. The data presented in Figure 3 and 4 were normalized to the maximum antigen expression measured in the absence of enzyme treatment. Statistical significance was assessed using appropriate significance tests (e.g., ANOVA) as implemented in GraphPad (GraphPad Prism Software, version 8.0.1). Significance levels are reported individually for each figure.

## Supporting Information

The authors have cited additional references within the Supporting Information.^[^ ^33^ ^,^ ^34^ ^]^

## Conflict of Interest

The authors declare no conflict of interest.

## Supporting information

Supplementary Material

## Data Availability

The data that support the findings of this study are available in the supplementary material of this article.
