# Supplementary material for: Immobilized Enzymes for ABO‐Independent Blood Cell Transfusions
Source: Chembiochem. 2025 Nov 23;26(24):e202500542. doi: 10.1002/cbic.202500542 (PMC12703440; doi:10.1002/cbic.202500542)
Supplement: Supplementary file 1 — Supplementary Material [file CBIC-26-e202500542-s001.pdf]

**Supporting information**

# Immobilized Enzymes for ABO-Independent Blood Cell Transfusions

*Christina Möller[1], Fabian Pohlschröder[2], Jan Wesche[2], Paula Schmiade[2], Merle Baselau[2], Kristian Wende[3], Berenika Stloukalová[1], Dominique Böttcher[1], Andreas Greinacher[2], Uwe T. Bornscheuer[1], Henrik Terholsen[1,†,*], Konstanze Aurich[2,*]*

[1]Institute of Biochemistry, Department of Biotechnology and Enzyme Catalysis

University Greifswald, 17487 Greifswald, Germany

[2]Institute of Transfusion Medicine, University Medicine Greifswald, 17475 Greifswald, Germany

[3]Leibniz Institute for Plasma Science and Technology, 17489 Greifswald, Germany

## 1. Methods

### Quality Control of Pooled Platelet Concentrates

*Platelet count.* Platelet count was measured in a 1:8 dilution with PAS-E by cell counter Sysmex XP-300 (Sysmex Deutschland GmbH, Norderstedt, Germany).

*Platelet function.* Platelet activation was determined by CD62P expression before and after the addition of thrombin receptor activating peptide 6 (TRAP-6). $3 x 10^8$ $mL^{-1}$ platelets were incubated for 10 min at 37 °C with 20 µM TRAP-6 (Hart Biologicals, UK) or phosphate buffered saline (PBS buffer; w/o $Ca^{2+}$, $Mg^{2+}$; pH 7.2) as the negative control, followed by addition of 5 µL 100 $\mu L^{-1}$ PE-Cy 5 Mouse Anti-Human CD62P (BD Bioscience, CA, USA) for additional 10 min. Samples were fixed 20 min with 0.5 % paraformaldehyde (PFA) and washed twice (2 mL PBS; 650 x g, 7 min, RT). The pellet was resuspended in 500 µL PBS and analyzed by flow cytometry (Cytoflex S, Beckman Coulter, USA). The increase of CD62P expression on platelets was determined using Pe-Cy5 mean fluorescence intensity (MFI) of the platelet-population given as fold increase in comparison to the respective buffer controls.

*Residual red blood cell count.* Residual red cells of PC units were counted using a Nageotte chamber (Assistent, Germany) under a microscope.

*Free hemoglobin.* Free hemoglobin, as a marker for hemolysis of red cells, was measured as cyanmethemoglobin spectroscopically using potassium hexacyanoferrate III and potassium cyanide in cell free supernatant. A multi wavelength absorption analysis was performed at 540 nm (A1) and 680 nm (A2) in a spectrophotometer (UV-1700, Shimadzu, Japan).

*Antibody binding on platelets.* Antibody binding to glycoprotein (GP) IIbIIIa, GP IbIX and Platelet Endothelial Cell Adhesion Molecule (PECAM-1; CD31) on platelet surfaces was determined by the monoclonal antibody immobilization of platelet antigens (MAIPA) assay.[33]

*Isoagglutinin titer.* Titers of anti-A and anti-B were determined by a microcolumn gel card system. For IgG titers cards containing six microcolumns with an anti-human globulin phase (ID-Card LISS/Coombs, Bio-Rad Laboratories Inc., USA), for IgM titers saline cards (ID-Card for sodium chloride, enzyme test and cold agglutinins, Bio-Rad Laboratories Inc., USA) were used according to the manufacturer's instructions. PC supernatant samples in 1:2 dilution series (dilution media: 0.9% (w/v) NaCl-solution) were incubated (IgG: 37 °C, 15 min; IgM: RT, 15 min) with group B red cells for anti-A and group A red cells for anti-B. Group O red cells were used as negative control. Agglutination strengths were evaluated by two independent individuals. Antibody titer was defined as the last sample dilution inducing agglutination.

**Protein Expression and Purification**

Synthetic genes for the galactosidases to investigate B antigen cleavage as well as synthetic genes for the enzymatic removal of A antigens were ordered in a pET28(a) vector (BioCat GmbH, Heidelberg, Germany). All genes contained an N- or C-terminal His-tag sequence for affinity chromatography. The sequences and NCBI accession codes are listed in Table S1.

For protein expression, the pET28(a) vectors harboring the synthetic genes were transformed into E. coli BL21 Gold (DE3) by the heat-shock method. In the case of each gene expression, a single colony of the cells with the desired amino acid sequence was picked and used to inoculate 4 mL LB media supplemented with 50 µg $mL^{-1}$ kanamycin, which was grown overnight at 37 °C at 140 rpm. These starter cultures were used to inoculate the 50 mL main cultures (TB medium)

in which the cells were grown until the optical density at 600 nm (OD600) reached approximately 0.6 - 0.8. The gene expressions of the cultures were induced with a final concentration of 0.5 mM isopropyl-β-D-thiogalactopyranoside (IPTG) and lasted around 20 hours at 20°C at 160 rpm. The main cultures were harvested by centrifugation (10 min, 2000 x *g,* 4 °C) and washed once with sodium phosphate buffer (50 mM, pH 7.5). The harvested bacteria pellets were resuspended in 4 mL washing buffer (50 mM sodium phosphate, 300 mM NaCl, 20 mM imidazole, 2 mM MgCl2, pH 8.0) for each gram of cell pellet. The cells were disrupted via ultrasonication with 30 % power and 50 % cycle on ice. Thereby, the sonication procedure consisted of 5 min sonication followed by a 2 min break and another 5 min of sonication. For separation of the cell debris from the supernatant, the samples were centrifuged at 10.000 x *g* for 30 min at 4 °C. The clarified lysates containing the desired proteins were transferred onto Ni-IDA columns (ROTI®Garose-His/Ni Beads, Carl Roth GmbH + Co. KG, Karlsruhe, Germany) for affinity chromatography, washed ten times with washing buffer and eluted in fractions with elution buffer (50 mM sodium phosphate, 300 mM NaCl, 2 mM MgCl2, 250 mM imidazole, pH 8.0). The fractions with the highest protein content were pooled and rebuffered in 50 mM sodium phosphate buffer pH 7.5 using Amicon Ultra-15 centrifugal filter units (MWCO 10 kD; Merck KGaA, Darmstadt, Germany). The protein solutions were stored at 4 °C until further use.

## Blood Group Antigen Determination on Blood Cells

*Red blood cells.* The effect of enzyme addition to RBC blood group antigens was determined by measuring the percentage of antigen A- and B-positive platelets by flow cytometry. RBC incubated with the enzymes were fixated like described by Otso et al.[34] In brief, 10 µL of RBC where incubatet with 10 µL of fixing solution (2 µL glutaraldehyde [GA, 25% aqueous solution] + 500 µl formaldehyd [FA, 4.21%]) for 10 min at RT and then washed twice with 120 µL PBS (w/o

Ca2+, Mg2+; PAN-Biotech, Germany; 1000 g for 5 min at RT). The pellet was finally resuspended in 120 µL PBS and the fixed RBC were incubated with 50 µL primary antibody Anti-A or Anti-B Monoclonal Immunoglobuline M (Optima Testseren, Bammental, Germany) for 15 min at RT. The samples were then washed twice with 120 µL PBS and the pellet was incubated with 50 µL of 1:10 diluted secondary antibody Polyclonal Rabbit Anti-Mouse Ig/FITC (Dako Deutschland GmbH, Germany) for 30 min at RT. Samples were washed once with 120 µL PBS and resuspended in 170 µL PBS for flow cytometry (see below).

*Platelets.* The effect of enzyme addition to platelet blood group antigens was determined by measuring the percentage of antigen A- and B-positive platelets by flow cytometry. 50 µL of platelets incubated with the enzyme solutions were fixated with 50 µL of 4 % paraformaldehyde (PFA, Morphisto Laborchemikalien, Offenbach am Main, Germany) and incubated for 15 min at RT and washed 2 times with 200 µL PBS (w/o $Ca^{2+}$, $Mg^{2+}$; PAN-Biotech, Germany) by centrifugation (7 min at 650 x g) and the pellet was finally resuspended in 50 µL PBS. 20 µL of the fixed platelets were incubated with 20 µL primary antibody Anti-A or Anti-B Monoclonal Immunoglobuline M (Optima Testseren,) for 20 min at RT. The sample was then washed twice and the pellet was incubated with 50 µL of 1:10 diluted secondary antibody Polyclonal Rabbit Anti-Mouse Ig/FITC (Dako Deutschland GmbH) for 30 min at RT. Samples were washed once and resuspended in 170 µL PBS.

All platelet and RBC samples were analysed by flow cytometry (Cytoflex S, Beckman Coulter, USA). Incubation of blood group A cells with Anti-B immunoglobuline and vice versa served as negative controls for background stain determination (Figure S6).

**Enzyme Immobilization on Microparticles**

Enzymes were immobilized on polymethacrylate ReliZymeTM size M 200 – 500 µm HFA 403 microparticles (MP, Resindion S.r.l., Italy). Covalent binding of the enzymes to the oxirane group on the particle shell was performed with 10 mg MP 2 $mL^{-1}$ sodium phosphate buffer (50 mM, pH 7.5) and ammonium sulfate (3 M) after two washing steps (650 x g; 3 min). MP were incubated at RT with either FpGalNAcDeAc and FpGalNase (final concentrations: 6 µM each) or PpaGal_WT, PpaGal_W260Y, AmGH110A, and AmGH110B (final concentration: 11.8 µM) overnight. Samples were washed twice with sodium chloride solution (0.5 M), incubated with glycine (1 M) and washed again with sodium phosphate buffer and stored at 4 °C.

*Enzyme amount immobilized on microparticles.* Samples were taken before and after 1, 2, and 4 hours of incubation to determine the protein concentration decrease in the supernatant (after centrifugation at 650 x g for 3 min) by UV/VIS spectrometry at 280 nm (Nanodrop 2000 Spectrophotometer, Thermo Fisher Scientific, Germany). We calculated the immobilization rate from the total enzyme amount applied to the sample before MP-addition minus the supernatant enzyme amount after the MP-addition.

**Impact of Enzyme Addition on Platelet Function**

The impact of the enzymes or microparticles addition to pooled PC was determined by the CD62P expression by flow cytometry as described above.

**Determination of Enzyme Residues in Platelet Concentrate Supernatant by Mass Spectroscopy**

Potential leakage of FpGalNAcDeAc (NCBI WP_009260926.1), FpGalNase (WP_044942952.1), and PpaGal (NQX53349.1) from beads into PC supernatant was determined using nano-Liquid Chromatography/Tandem Mass Spectrometry (LC-MS/MS). Sample

preparation: A fraction of the supernatant containing 250 µg of proteins was diluted 1:5 in PBS w/o $Ca^{2+}/Mg^{2+}$, and precipitated in 80% acetone at -20°C overnight. The precipitate was spun down for 5 min at 5000 g, washed once with 80% acetone, air-dried for 5 min and dissolved in 50 mM ammonium bicarbonate buffer (pH 8.0). Reduction was achieved by adding a final concentration of 10 mM dithiothreitol and incubation at 60°C for 30 min, followed by alkylation by iodoacetamide (10 mM final concentration, 20 min at RT). For digestion, sequencing-grade trypsin (Promega, V5111) was used in a ratio of 1:40 at 37°C overnight. The reaction was stopped by reducing the pH to 1 by adding HCl. For desalting and clean-up, sample was subjected to C18 solid phase extraction (Luna C18 3um, Phenomenex, Aschaffenburg, Germany). Peptides were diluted by 0.1% formic acid in 80% of acetonitrile in water (uHPLC-MS quality, Thermo Fisher Scientific, Waltham, USA). The eluate was dried in a SpeedVac at room temperature and reconstituted in 10 µl 0.1% formic acid in water (Buffer A). For mass spectrometry analysis, 500 ng peptide was loaded onto a PepMap C18 trap column (5 µm particles, 20 x 0.1 mm) and separated on a PepMap C18 analytical column (3 µm particles, 150 x 0.075 mm; Dionex UltiMate 3000 RSLCnano) using buffer A and 0.1 % formic acid in 95:5 ACN:MS grade water (buffer B) at a flow rate of 300 nL min-1 at 40°C. A linear gradient of 2 to 35 % buffer B over 75 min was used. Analytes were ionized by electrospray in positive mode (+2.4 kV) using a metal emitter and transferred into an Exploris 480 orbitrap mass analyser (Thermo Fisher Scientific, Waltham, USA). The mass spectrometer was operated in data dependent acquisition mode (DDA). MS1 full scan parameters were set as follows: 350–1200 m z-1, R = 120,000 at 200 m $z^{-1}$, target of 5 × 103 ions, followed by up to 15 data-dependent MS/MS scans with higher energy collision dissociation (HCD, maximum injection time (IT) 50 ms, isolation width 1.0 m z-1, NCE 30%, R = 15,000 at 200 m z-1). Dynamic exclusion was enabled and set to 30 s. For quantification, the instrument was

used in data independent acquisition mode (DIA), a full MS1 spectrum was recorded for 390–1010 m z-1, R = 15,000 at 200 m z-1, target of 5 × 103 ions, followed by 60 MS2 scans with each 10 m z-1 isolation window, spanning from 399.5 m z-1 to 1000.5 m z-1 with a fixed injection time of 22 ms and a resolution of R = 15,000 at 200 m z-1. DDA raw data analysis was achieved by ProteomeDiscoverer 2.5 (Thermo Fisher Scientific, Waltham,USA), using a hybrid fasta file containing the sequences of FpGalNAcDeAc/FpGalNase, and PpaGal (Table S2) and the human proteome (uniprot.org, Sep 2023, 26255 sequences), yielding to the unambiguous detection of FpGalNAcDeAc, FpGalNase or PpaGal in control samples with 32, 38, and 38 unique peptides, respectively. Information of identified peptides was subsequently used for relative quantification

of FpGalNAcDeAc, FpGalNase and PpaGal in DIA raw data by Skyline 21.2.0.565 (MacCoss Lab, University of Washington, USA) within the human proteome as the background. For each protein, three unique peptides showing best performance regarding quality and numbers of detection in control samples were selected. To visualize and summarize the results the areas of the precursor ions M, M+1, M+2 (natural $^{13}C$ isotope containing variants) and the related fragment ions (mainly y-ions) of the tryptic peptides DLVASGSDWALDAK (597 - 610, FpGalNAcDeAc), TDEAGAYAELTFR (865 – 877, FpGalNase), and NTTPLLTSLLK (55 – 65, PpaGal) are shown. Unless otherwise stated, all consumables were purchased from Thermo Fisher, Waltham, USA.

**Statistics and Reproducibility**

The data are shown as mean with standard error of mean. All replicates are biological replicates from distinct samples. GraphPad Prism 8.0.1 software (GraphPad Software, La Jolla, CA) was used for statistical analysis. Samples were tested for lognormal distribution by Shapiro-Wilk-test. When normal distributed ordinary one way ANOVA followed by Fishers LSD Test was used. Data

with only two comparable data sets were analyzed by paired t-test. P-values <0.05 were considered to be statistically significant: *P<0.05, **P<0.01, ***P<0.001, and ****P<0.0001.

## 2. Supporting Figures

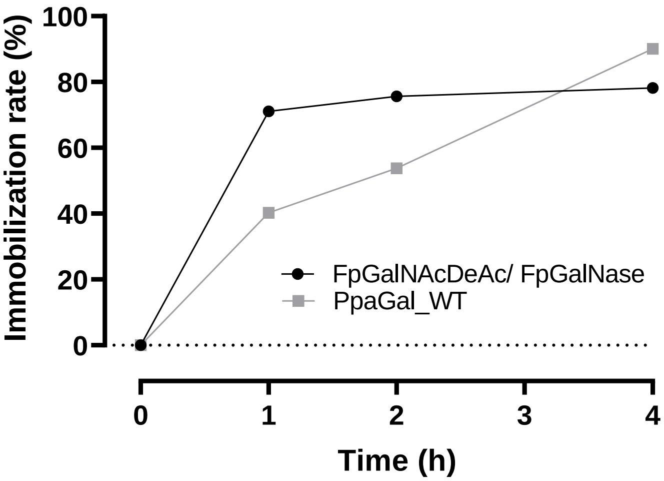


**Figure S1.** Immobilization rate of FpGalNAcDeAc and FpGalNase (incubation concentration 6 µM) and representative galactosidase PpaGal_WT (incubation concentration 11.8 µM) on RelizymeTM HF403 microparticles within 4 hours incubation time (n=2, mean).

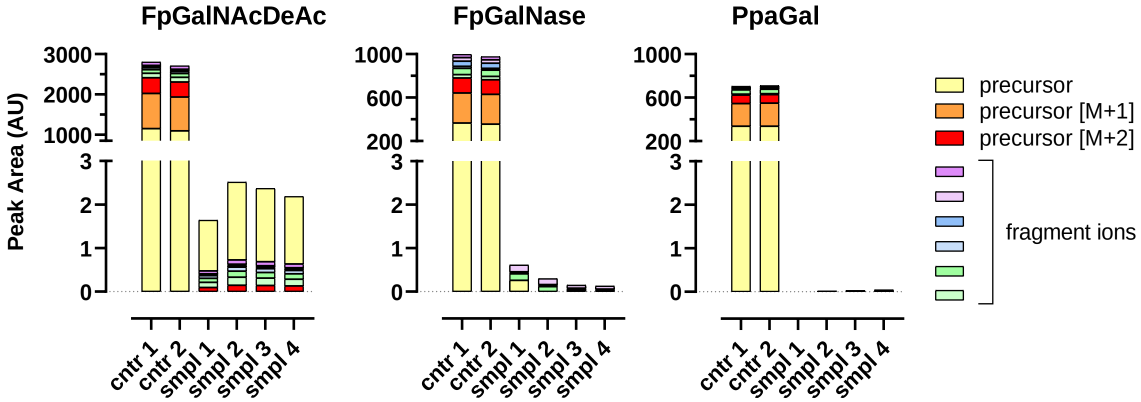


**Figure S2.** Minor leakage of FpGalNAcDeAc-ReliZymeTM HF403 from microparticles into PC supernatant was detected, but not for FpGalNase and PpaGal as confirmed by nanoLC-MS/MS. Bars show peak areas for precursor and related fragment ions of one major unique peptide (DIA mode raw data, see methods section). One control (cntr) or two samples (smpl) technical replicates were injected twice.

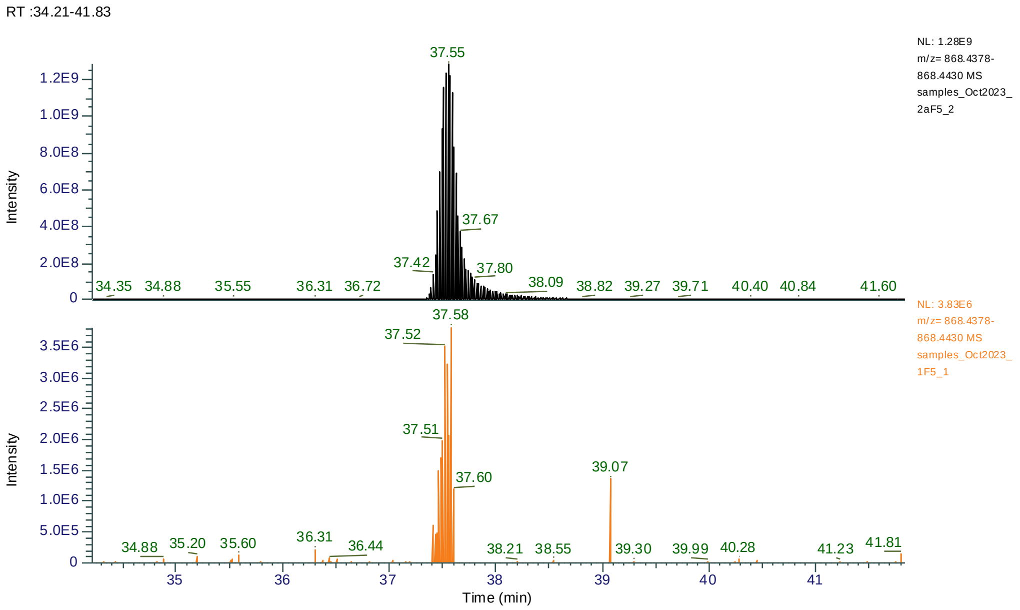


**Figure S3.** Extracted ion chromatogram (XIC) for control (top) or sample (bottom) of FpGalNAcDeAc (P0DTR4), peptide LETSFAPVDTSDQVVK [443, 458]. Note the different ranges of the y-axis.

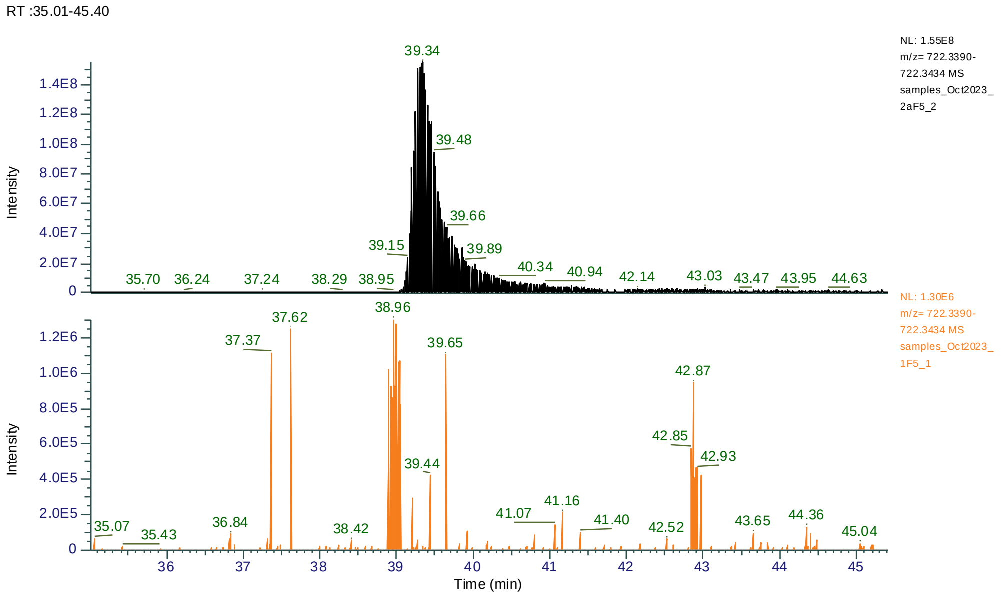


**Figure S4.** Extracted ion chromatogram (XIC) for control (top) or sample (bottom) of FpGalNase (P0DTR5), peptide TDEAGAYAELTFR [865 - 877]. Peptide was not detected in the sample.

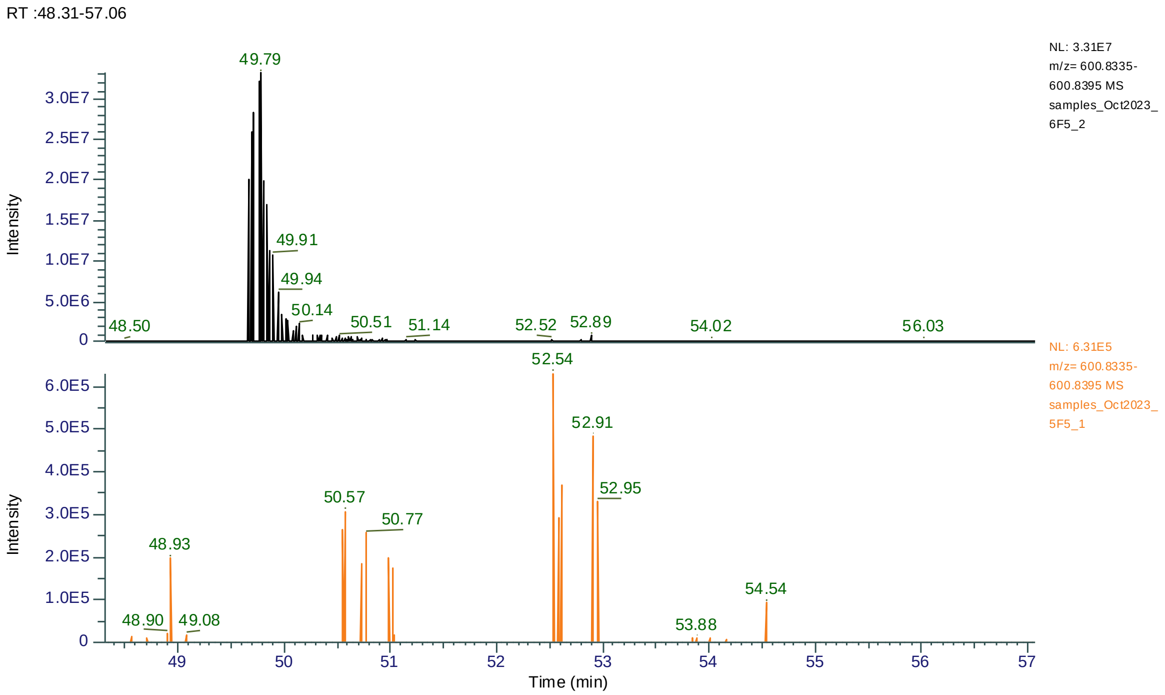


**Figure S5.** Extracted ion chromatogram (XIC) for control (top) or sample (bottom) of PpaGal (W6TP58), peptide NTTPLLTSLLK [55 - 65]. Peptide was not detected in the sample.

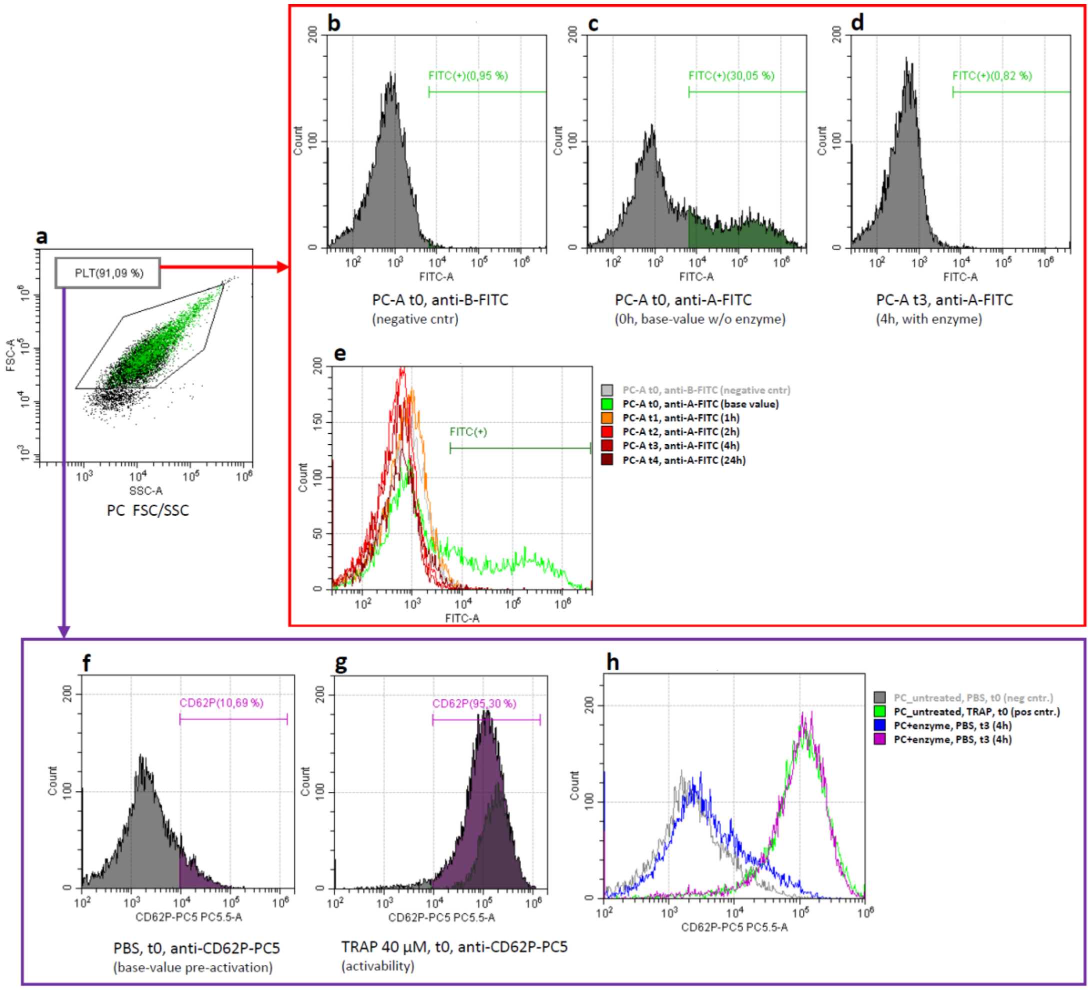


**Figure S6.** Flow cytometry gating and analysis of platelets (PLT) blood groups (B-E) or CD62P activation status (F-H). **a)** PLT population was determined by size in the forward/sideward scatter blot (FSC/SSC) and analyzed for binding of primary mouse anti-blood-group-antibodies followed by secondary FITC-conjugated anti-mouse-IgG (B-E) or direct binding of PeCy5-conjugated CD62P activation marker (F-H).

**b)** representative blood group-A platelet concentrates (PC-A) at timepoint t0 (before addition of enzymes) incubated with primary anti-B-antibody as negative control to set the background stain. **c)** PC-A at t0 incubated with primary anti-A-antibody to set the base-value of group-A positive platelets (this value was normalized to reflect 100% A-positive PLT), and **d)** PC-A at t3 (4h after enzyme addition). **e)** blood-group stain histogram overlay of negative control (grey), base-value (green) and all 4 timepoints after enzyme incubation (t1=1h, t2=2h, t3=4h, t4=24h; light to dark-red).

**f-g)** representative CD62P histograms of untreated PC before (F) and after (G) addition of 40 µM TRAP-6 at timepoint t0. **h:** CD62P-activation histogram overlay of untreated PC (grey+green) and after 4h of enzyme incubation (blue+purple), each with buffer (PBS) or 40 µM TRAP-6.

## 3. Supplementary Tables

**Table S1.** Isoagglutinin titer and buffy coat blood groups of pooled platelet concentrates from buffy coats of non-identical blood groups.

| | IgG | | IgM | | |
|---|---|---|---|---|---|
| **Platelet concentrate No** | **anti A** | **anti B** | **anti A** | **anti B** | **Buffy coat blood groups** |
| 1 | <1:1 | 1:16 | <1:1 | <1:1 | A A O O |
| 2 | <1:1 | <1:1 | <1:1 | <1:1 | A AB O B |
| 3 | <1:1 | <1:1 | <1:1 | <1:1 | A AB O B |
| 4 | <1:1 | <1:1 | <1:1 | <1:1 | A O B B |
| 5 | <1:1 | <1:1 | <1:1 | <1:1 | A AB O B |
| 6 | <1:1 | <1:1 | <1:1 | <1:1 | A AB O O |
| 7 | <1:1 | <1:1 | <1:1 | <1:1 | A AB O O |
| 8 | <1:1 | 1:8 | 1:16 | 1:16 | A A O O |
| 9 | <1:1 | <1:1 | <1:1 | <1:1 | A A AB O |
| 10 | <1:1 | <1:1 | <1:1 | <1:1 | A A B B |
| 11 | <1:1 | <1:1 | <1:1 | <1:1 | AB A A B |
| 12 | <1:1 | <1:1 | <1:1 | <1:1 | AB O B O |

**Table S2.** Target protein characteristics and mass spectrometry results from complex blood serum background (477 proteins, 192,440 PSMs*)

| **NCBI code** | **Protein name** | **Cove-rage (%)** | **No. of peptides** | **Global no. of PSMs*** | **No. of AAs** | **MW (kDa)** | **DDA (PD 2.5)§** | **DIA (Skyline 21.2.0.565)&** |
|---|---|---|---|---|---|---|---|---|
| **WP_009260926.1** | *N*-acetyl-α-D-galactosamine deacetylase | 71 | 32 | 2623 | 780 | 85.5 | 10 / 25 (0.95 %) | yes - 0.55 µg mL-1 |
| **WP_044942952.1** | α-D-galactosaminidase | 55 | 38 | 2034 | 1086 | 119.7 | 0 / 0 | n.d. |
| **NQX53349.1** | α-1,3-galactosidase B | 69 | 38 | 579 | 617 | 69.2 | 1 / 1 | n.d. |

* PSM = Peptide spectra match (unique fragment pattern identifying the peptide), number correlates positively with the protein concentration in the analyte, number of PSMs found in control and test samples is given D

$ DDA = Data dependent analysis approach; PD = ProteomeDiscoverer, data represent number of peptides / number of PSMs detected from test samples and attributed to the target protein

& DIA = data independent analysis approach; column specifies if, and in which estimated amount the target proteins could be detected in test sample; n.d. – no precursor or fragment ions detected for the target protein

**Amino acid sequences and NCBI codes of the synthetic genes of the investigated enzymes containing an N-terminal or C-terminal His-tag and no signal peptide that were ordered in pET28a(+) vector.**

*B antigen removing enzymes:*

*PpaGal (α-1,3-galactosidase from* Pedobacter panaciterrae*, based on NCBI-Code: NQX53349.1):*
MGSSHHHHHHSSGLVPRGSHMASMTGGQQMGRGSEFNVKIYKLSAYGIKPNSGKNTTPLLTSLLKEIKSKT
SDLDKVIIQFEKGRYDFYPEGAIKREYYISNHDQDNPKTVGIGIEKFNNITLIGKGTDLMFHGRMLPLALIESS
NVKIKDLNIDFEKPQITQVKIISNDTTAGNIVFETAPWVKYKLKDSTFYNTGEGWEMQPTSGIAFENGTKHII
FNSGDIGVGTKSVSEVSPGKIMAHHWKNKKLVPGTVIAMRSWQRPAPGIFVHKGKNISFENVKVHYAEGM
GLLAQLTENIYMDGFGVCLRGKNDPRYFTTQADATHFSGCKGEIVSKNGLYEGMMDDAINIHGTYLKITK
KLDDHTVIANYMHEQSYGFDWGNIRDTVQFIQSKTMELWDAKNTIASIKPILRNSTDPIKEFRIEFTKALDP
VIDPSKQDIGIENLSWTPSVVFTGNTIRNNRARGALFSTPKPTLVANNLFDHTSGCAILLCGDSNGWYETGS
CRDITIRDNKFVNALTSMYQFTSAIISIYPEIPDLTNQKKYFHSGIRILNNQFDTFDQPILYAKSVDGLVFTGN
KIQTNKEYPAFHSNKKRFLFERVIGVDFSDNKVDGKPIEML

*PpaGal_W260Y (α-1,3-galactosidase from* Pedobacter panaciterrae*, based on NCBI-Code: NQX53349.1):*
MGSSHHHHHHSSGLVPRGSHMASMTGGQQMGRGSEFNVKIYKLSAYGIKPNSGKNTTPLLTSLLKEIKSKT
SDLDKVIIQFEKGRYDFYPEGAIKREYYISNHDQDNPKTVGIGIEKFNNITLIGKGTDLMFHGRMLPLALIESS
NVKIKDLNIDFEKPQITQVKIISNDTTAGNIVFETAPWVKYKLKDSTFYNTGEGWEMQPTSGIAFENGTKHII
FNSGDIGVGTKSVSEVSPGKIMAHHWKNKKLVPGTVIAMRSYQRPAPGIFVHKGKNISFENVKVHYAEGM
GLLAQLTENIYMDGFGVCLRGKNDPRYFTTQADATHFSGCKGEIVSKNGLYEGMMDDAINIHGTYLKITK
KLDDHTVIANYMHEQSYGFDWGNIRDTVQFIQSKTMELWDAKNTIASIKPILRNSTDPIKEFRIEFTKALDP
VIDPSKQDIGIENLSWTPSVVFTGNTIRNNRARGALFSTPKPTLVANNLFDHTSGCAILLCGDSNGWYETGS
CRDITIRDNKFVNALTSMYQFTSAIISIYPEIPDLTNQKKYFHSGIRILNNQFDTFDQPILYAKSVDGLVFTGN
KIQTNKEYPAFHSNKKRFLFERVIGVDFSDNKVDGKPIEML

*AmGH110A (α-1,3-galactosidase from* Akkermansia muciniphila*, based on NCBI-Code: CDB55591):*

MGSSHHHHHHSSGLVPRGSHMASMTGGQQMGRGSEFEEKAAQSGTIAVKVPASSLLMTRQETGETRLDR
SFSNAGLSIGGKKYATGIGTHATSMIPLPVPENPKVLRLEGACGIDDGADGDGSVEFRVMSGSEVLWSSGV
MRRGMAAKKFSIPVAENGIRHLYLMADRVDNNSYDHADWVDLAWKTTGSGQGMKGAVVNASEFGMVP
GVRKDQGPALRAAVSALRRQGGGVLNIPRGIYHFYPEGALNMSFHISNHDQPLIHPVCVPLADLRNVRVEG
NGSLFLFHGKVVPLLVMDSENVSINRLSVDYERSWCTEARVVKTDDRFTEVEIDKKAYPYEIRNNRFVFQG
KGWEEGMGSCMAFEKGTGHIIANTSDIGWNGHVEPLGGSRLRLSWNLRQKGIKPGDTLVLRNYNRPHPGC
VVYRARKTSLNDVSLHQSSGMALLVQRSEDFHMKGGGVMVRKGTGRVHTAGADATHFSNTRGGIVVEK
ALFEGMMDDAINVHSTCLGVMEVVDSHTLKCKYMHRQAVGFEVFLPGEKIRFINGPTLEPGGTATVKTAV
KKNSAEMVITVEEPLPSSVRAGDAVENADFYPSVVFRNNIVRNNRARGSLFTTPERVLVEGNLFDHSSGSAI
LLAGDAQGWYESGACHEVVIRKNTFINNLTSRYQFTNAIISIYPEVKQLDRQRDYYHRNVLIENNVFKTFDV
PLLFAISTDNLKFINNKVIYNDEFKGWGQKPFQFRRCANILIKDNKVLPPRTWTLEDCKLENTPSDQVRFGG

*AmGH110B (α-1,3-galactosidase from* Akkermansia muciniphila*, based on NCBI-Code: ACD05285):*

MGSSHHHHHHSSGLVPRGSHMASMTGGQQMGRGSEFADYPERTERTQSAGNHVWHIDPDKGNDGNPGT
APSTAWKSMAPANRLIMARGDTLVIHPGEHAVSLALMGEGSKQAPVTIRFMPGRHIFKHGALMTGKPQISN
TNDAPNEPKAMAIRLMEAKNIRLEGKPGATDILLEGKAIFVCMEHAENVSLNGLGFDYLHPTMGEFLVTEV
EGDTMKATIPDGTLYTVKDGNLTWHGPGWEFRMGGYSKVFDSASGTFQGRFDPGKTVIRELSPGKISITFK
EGSPTMKPGQSYQNRNTRRDCCGFFQYRSKNILWNNCHIYYMHGMGVVSQFCENIMFSHLKIAPRPRSLRT
NSSWADNLHFSGCRGKIIVKDCVLGASHDDAVNVHGTHLRIIDRPAPNKITVRFMHPQTFGFDAFAAGDRI
DYVSCNTLVPYASNTVSGVKQLNEKEIELTLQHPNPGNIQPDDVVENVTWTPSVHISNTVCRHIPTRGFLLT
TRKPVLVERCRFEKTGMPAILVEDDASGWYESGVVRNMTISRNTFIQCGEAVIQIVPHAPRPEGDVHRNITI
TGNTFDLKNGTAIRIRHTGDVKAEKNTFTKDGKKIPEEKAVDIR

*A antigen removing enzymes:*

*FpGalNAcDeAc (N-acetyl-α-D-galactosamine deacetylase from* Flavonifractor plautii*, based on NCBI-Code: WP_009260926.1):*

MADSSESALNKAPGYQDFPAYYSDSAHADDQVTHPDVVVLEEPWNGYRYWAVYTPNVMRISIYENPSIV
ASSDGVHWVEPEGLSNPIEPQPPSTRYHNCDADMVYNAEYDAMMAYWNWADDQGGGVGAEVRLRISYD
GVHWGVPVTYDEMTRVWSKPTSDAERQVADGEDDFITAIASPDRYDMLSPTIVYDDFRDVFILWANNTGD
VGYQNGQANFVEMRYSDDGITWGEPVRVNGFLGLDENGQQLAPWHQDVQYVPDLKEFVCISQCFAGRNP
DGSVLHLTTSKDGVNWEQVGTKPLLSPGPDGSWDDFQIYRSSFYYEPGSSAGDGTMRVWYSALQKDTNN
KMVADSSGNLTIQAKSEDDRIWRIGYAENSFVEMMRVLLDDPGYTTPALVSGNSLMLSAETTSLPTGDVM
KLETSFAPVDTSDQVVKYTSSDPDVATVDEFGTITGVSVGSARIMAETREGLSDDLEIAVVENPYTLIPQSN
MTATATSVYGGTTEGPASNVLDGNVRTIWHTNYAPKDELPQSITVSFDQPYTVGRFVYTPRQNGTNGIISE
YELYAIHQDGSKDLVASGSDWALDAKDKTVSFAPVEAVGLELKAIAGAGGFGTAAELNVYAYGPIEPAPV
YVPVDDRDASLVFTGAWNSDSNGSFYEGTARYTNEIGASVEFTFVGTAIRWYGQNDVNFGAAEVYVDGV
LAGEVNVYGPAAAQQLLFEADGLAYGKHTIRIVCVSPVVDFDYFSYVGELEHHHHHH

*FpGalNase (α-D-galactosamine galactosaminidase from* Flavonifractor plautii*, based on NCBI-Code: WP_044942952.1):*

MAAPATDTGNAGLIAEGDYAIAGNGVRVTYDADGQTITLYRTEGSGLIQMSKPSPLGGPVIGGQEVQDFSH
ISCDVEQSTSGVMGSGQRMTITSQSMSTGLIRTYVLETSDIEEGVVYTATSYEAGASDVEVSWFIGSVYELY
GAEDRIWSYNGGGEGPMHYYDTLQKIDLTDSGKFSRENKQDDTAASIPVSDIYIADGGITVGDASATRREV
HTPVQETSDSAQVSIGWPGKVIAAGSVIEIGESFAVVHPGDYYNGLRGYKNAMDHLGVIMPAPGDIPDSSY
DLRWESWGWGFNWTIDLIIGKLDELQAAGVKQITLDDGWYTNAGDWALNPEKFPNGASDALRLTDAIHE
HGMTALLWWRPCDGGIDSILYQQHPEYFVMDADGRPARLPTPGGGTNPSLGYALCPMADGAIASQVDFV
NRAMNDWGFDGFKGDYVWSMPECYNPAHNHASPEESTEKQSEIYRVSYEAMVANDPNVFNLLCNCGTP
QDYYSLPYMTQIATADPTSVDQTRRRVKAYKALMGDYFPVTADHNNIWYPSAVGTGSVLIEKRDLSGTAK
EEYEKWLGIADTVQLQKGRFIGDLYSYGFDPYETYVVEKDGVMYYAFYKDGSKYSPTGYPDIELKGLDPN
KMYRIVDYVNDRVVATNLMGDNAVFNTRFSDYLLVKAVEISEPDPEPVDPDYGFTSVDDRDEALIYTGTW
HDDNNASFSEGTARYTNSTDASVVFSFTGTSIRWYGQRDTNFGTAEVYLDDELKTTVDANGAAEAGVCLF

EALDLPAAEHTIKIVCKSGVIDIDRFAYEAATLEPIYEKVDALSDRITYVGNWEEYHNSEFYMGNAMRTDE
AGAYAELTFRGTAVRLYAEMSFNFGTADVYLDGELVENIILYGQEATGQLMFERTGLEEGEHTIRLVQNA
WNINLDYISYLPEQDQPTPPETTVTVDAMDAQLVYTGVWNDDYHDVFQEGTARYASSAGASVEFEFTGSE
IRWYGQNDSNFGVASVYIDNEFVQQVNVNGAAAVGKLLFQKADLPAGSHTIRIVCDTPVIDLDYLTYTTN
ALEHHHHHH
